# A Pageant for Hospitals

**Published:** 1921-12

**Authors:** 


### A PAGEANT FOR HOSPITALS.

At a meeting of representatives of the hospitals of London, held at the Mansion House on November 28, the Earl of Bessborough in the chair, the question of making a combined effort next year on behalf of the Metropolitan hospitals was considered. The suggestion is that arrangements shall be made for holding a Hospital Week and Pageant in May quite distinct from existing institutions like Hospital Sunday.

Mr. G. H. Hamilton explained that the matter had been under consideration for some time. The view, however, was that it would be no use organising a Hospital Week and Pageant unless the large majority of the hospitals gave their support, and that no scheme could be worked except in complete harmony with the King's Fund. For some time there had been a general desire for a joint effort and appeal rather than individual spasmodic efforts. A Hospital Week was new to this country, but something similar had been attempted by the United States and Canada. In New York last year the hospitals were thrown open to the general public for a period, and about 250,000 people availed themselves of the opportunity of inspecting them. One of the results was that a large number of recruits joined the nursing staffs of the hospitals. The period suggested in London was from May 8 to May 14 next—the pageant to parade the north side of the river on one day and the south side on another; that there should be a King's Day; and that on the other days the public should be invited to visit the hospitals. It was hoped that on King's Day it might be possible to get His Majesty to show some mark of appreciation. It was suggested that collections should be made in the streets, and that various tradesmen might be induced to place hospital exhibits in their windows. He did not think that the scheme would be a costly one. Probably £4,000 would cover everything. Expert opinion as to the probable cost had been obtained. They might hope to raise £50,000.

Mr. Pentney (Metropolitan Hospital) said that last year the Metropolitan had a carnival and £1,000 was raised. This year the amount was £1,500, and the expenses were about 8 per cent. Arrangements had been made to run a carnival next year, and the date had been practically fixed for June. He did not wish to be considered selfish, but one had to consider the interests of one's hospital a little. Supposing £40,000 was raised; deducting £4,000, £36,000 would be left, and that sum divided among seventy hospitals would mean less than £600 for each. Therefore it was questionable whether the scheme would be worth while.

Mr. E. Eason (Guy's Hospital) thought that the scheme should be left to the King's Fund for their approval or disapproval. The real question to be decided was whether the return would make it worth while spending £4,000.

Mr. Elliott (of the King's Fund) explained that the scheme was being considered by the King's Fund in conjunction with the Regional Committee, and he hoped a decision would be reached before long.

Other speakers expressed the view that local efforts for particular hospitals should be continued apart from the proposed joint appeal.

On the motion of the Chairman, the following resolution was carried, it being understood that after a decision had been come to by the King's Fund and Regional Committee another meeting should be called:

"This meeting approves of the recommendation made by the Committee of Hospital Secretaries for the holding of a Pageant and Hospital Week for the benefit of the hospitals of London, subject to the following conditions: (a) Agreement to support the scheme being received from sufficient boards of governors to represent 80 per cent. of the bed accommodation and (or) out-patient attendances of London voluntary hospitals; (b) the project being approved by and carried out under the auspices of King Edward's Hospital Fund for London, which is primarily concerned with the special effort to co-ordinate hospital propaganda."

---

On November 14 the Association of Certificated Blind Masseurs held its third annual dinner in the King's Room of Pagani's Restaurant. The President of the Association, Sir Arthur Pearson, Bart., G.B.E., was in the chair. The gathering was a representative one, the members present including soldiers blinded in the war and trained in massage at St. Dunstan's, and civilian masseurs and masseuses trained under the auspices of the National Institute for the Blind.

